# Consumption of minimally processed foods as protective factors in the genesis of squamous cell carcinoma of the head and neck in Brazil

**DOI:** 10.1371/journal.pone.0220067

**Published:** 2019-07-25

**Authors:** Olívia Perim Galvão De Podestá, Stela Verzinhasse Peres, Luciane Bresciani Salaroli, Monica Cattafesta, José Roberto Vasconcelos De Podestá, Sandra Lúcia Ventorin von Zeidler, José Carlos de Oliveira, Luiz Paulo Kowalski, Mauro Kasuo Ikeda, Paul Brennan, Maria Paula Curado

**Affiliations:** 1 Postgraduate Program in Sciences of Fundação Antônio Prudente, Cancer Center of A.C.Camargo, São Paulo—SP / BR; 2 Graduate Program in Collective Health, Federal University of Espírito Santo, Vitória—ES / BR; 3 Women's Cancer Association Santa Rita de Cássia Hospital, Vitória—ES / BR; 4 Postgraduate Program in Biotechnology, Federal University of Espírito Santo, Vitória—ES / BR; 5 Association to Combat Cancer in Goiás Hospital Araújo Jorge, Goiânia—GO / BR; 6 International Agency for Research on Cancer, Lyon, France; National Health Research Institutes, TAIWAN

## Abstract

**Background:**

Head and neck cancer (HNC) is the sixth most common cancer, and two-fifths of cases could be avoided by changing lifestyle and eating habits.

**Methods:**

This multicenter case-control study was conducted under the International Consortium on Head and Neck Cancer and Genetic Epidemiology, coordinated by the International Agency for Research on Cancer. This consortium evaluated associations between minimally processed food consumption and the risk of HNC in three Brazilian states.

**Results:**

We evaluated 1740 subjects (847 cases and 893 controls). In multiple analyses including recognized risk factors for HNC, the consumption of apples and pears was associated with reduced risks of oral cavity and laryngeal cancers; the consumption of citrus fruits and fresh tomatoes was associated with a reduced risk of oral cavity cancer; the consumption of bananas was associated with a reduced risk of oropharynx cancer; the consumption of broccoli, cabbage, and collard greens was associated with reduced risks of laryngeal and hypopharyngeal cancers; and the consumption of carrots and fresh fruits was associated with a reduced risk of hypopharyngeal cancer.

**Conclusions:**

The consumption of a heathy diet rich in fruits and vegetables was associated with a reduced risk of HNC. Public policies, including government subsidies, are essential to facilitate logistical and financial access to minimally processed foods, thereby strengthening environments that promote healthy behavior.

## Introduction

Head and neck cancer (HNC) is the sixth most common cancer in the world, accounting for approximately 6% of all malignancies [1]. The National Cancer Institute of Brazil predicts the occurrence of approximately 31,980 HNC cases in each year of the 2018–2019 biennium, including 14,700 cases in the oral cavity and 7670 cases in the larynx, both of which are more frequent in men [2].

Risk factors for cancers of the mouth, larynx, oropharynx, and hypopharynx include alcohol consumption, smoking, human papillomavirus infection, and a nutrient-poor diet [3–9]. Approximately two-fifths of the most common neoplasms may be avoided by lifestyle and dietary modifications [10], and the combination of smoking and alcohol consumption is responsible for almost 80% of the risk of HNC [11]; however, diet is important in the genesis of these tumors, as certain foods play protective roles due to their bioactive compounds and modulators [11–15]. Vegetables and fruits provide micronutrients, dietary fiber, and phytochemicals [8,10], reducing the risk of malignant neoplasias of the oral cavity, larynx, and pharynx [13–15] due to their regulation of the expression and activity of transcription factors, growth factors, inflammatory mediators, and cell cycle intermediates [16].

Minimally processed foods are viewed as protective and include fruits, raw greens, vegetables, roots, seeds, and tubers, as well as some animal products. They are classified according to the extent of processing to increase shelf and storage lives, facilitate or diversify cooking preparation, and/or modify flavor [17]. This food group, according to the Food Guide for the Brazilian Population, should be the basis of a nutritionally balanced, tasty, culturally appropriate diet and for the promotion of a socially and environmentally sustainable food system [17,18]. Thus, the objective of this study was to evaluate minimally processed food consumption and the risk of developing cancer of the oral cavity, larynx, oropharynx, and hypopharynx.

## Material and methods

This multicenter case-control study was part of the International Consortium on Head and Neck Cancer and Genetic Epidemiology Study coordinated by the International Agency for Research on Cancer. Patients were recruited from July 2011 to July 2017 from the Association to Combat Cancer at the Goiás Hospital Araújo Jorge (Goiânia, GO), the A.C. Camargo Cancer Center Hospital (São Paulo, SP) and the Women's Association to Combat Cancer at Santa Rita de Cássia Hospital (Vitória, ES). The study was approved by the Committee on Ethics in Research in Human Beings of the Araujo Jorge Goiania Hospital and the Antônio Prudente Foundation Cancer Hospital of A.C. Camargo Cancer Center (no. 1670/12b), and by the National Council of Research Ethics (no. 16525/2011). The research followed the precepts of the Declaration of Helsinki, and all participants provided written informed consent.

### Inclusion and exclusion criteria

Patients diagnosed with epidermoid carcinoma of the oral cavity, larynx, oropharynx, or hypopharynx who were 18–80 years of age were considered to be eligible. Patients treated previously for cancer, those with more than one tumor, and those with clinical and mental conditions preventing them from responding appropriately to the questionnaire were excluded. All cases of squamous cell carcinoma of the oral cavity, larynx, oropharynx, and hypopharynx were confirmed by histology and classified according to the third revision of the International Classification of Diseases for Oncology [19].

### Case-control matching

Cases were paired independently with controls according to age and sex. The cases and controls were paired at a ratio of 1:2 for cancers of the oral cavity, oropharynx, and larynx, and at a ratio of 1:3 for hypopharyngeal cancer. Individual participating centers selected hospital or population sources of the controls. Controls in São Paulo were recruited from cancer prevention campaigns, those in Goiânia were patients recruited from hospitals in the state public network that did not specialize in oncology, and controls in Vitoria were non-consanguine companions of patients with cancer. The hospital controls were selected from patients with a restricted set of chronic diseases and other conditions not associated with alcohol or tobacco use (endocrine/metabolic, genitourinary, skin, subcutaneous/musculoskeletal tissue, gastrointestinal, eye/ear/mastoid, and nervous system diseases; trauma; circulatory disorders; indications for minor or plastic surgery; low back pain; and urinary tract infections).

### Covariates

The sociodemographic characteristics recorded were self-reported race/skin color ("white" and "non-white") and education level ("illiterate," "elementary school," "high school," and "higher education"). Sex and age group ("less than 60 years" and "greater than or equal to 60 years") were paired variables.

Alcohol consumption was classified as "never drank," "former drinker," and "current drinker." Smoking was classified as "never smoked," "former smoker," and "current smoker."

Oral hygiene was investigated by physical exam (by a trained professional, dentist or head and neck surgeon) and categorized as "good," "reasonable," or "bad," and nutritional status was characterized using the body mass index [BMI; weight (kg) / height (m)^2^]. For adults (18–59 years), BMIs < 18.5 kg/m^2^ were classified as low, BMIs of 18.5–24.9 kg/m^2^ were classified as normal, and BMIs ≥ 25 kg/m^2^ were taken to indicate overweight [20]; for patients aged > 60 years [21], these categories were defined by BMIs < 23 kg/m^2^, 23.0–28.0 kg/m^2^, and >28 kg/m^2^, respectively.

The habit of consuming minimally processed foods was evaluated according to participants’ reported consumption of the following food items/groups: all vegetables (except potatoes), with the subgroups of raw greens and vegetables; broccoli, cabbage, and collard greens (cruciferous vegetables); and carrots; and all fresh fruit items, with the subgroups of natural fruit juices; apples and pears; citrus fruits (oranges, lemons, and tangerines); fresh tomatoes; and bananas; and rice and beans. The frequency of consumption of these foods was classified as "never or <once a month," "1 to 3 times a month," "1 to 2 times a week," "on most days but not every day," and "every day"; for patients with hypopharyngeal cancer, consumption was classified as "<3 times a month," "1 to 2 times a week" and "almost every day or more" for multiple modeling. The consumption of rice and beans was classified as "do not consume rice and beans or consume little," "consume rice and consume little beans," "consume little rice and consume more beans," and "consume rice and beans almost daily."

### Statistical analysis

The data were analyzed descriptively by calculating means of absolute and relative frequencies, and applying measures of central tendency and dispersion. The chi-squared test was applied to verify associations between the covariates (sociodemographic, clinical, lifestyle, and nutritional characteristics) and the outcome of cancer. The endpoints considered were the presence of oral cavity, oropharyngeal, laryngeal, and hypopharyngeal cancer, respectively.

Univariate binary logistic regression analysis (S1 Table) and unconditional binary multiple logistic regression analysis were performed to obtain odds ratios (ORs) with 95% confidence intervals (CIs). Covariables that presented significant *p* values (<0.050) and those with *p* values < 0.200 were included in multiple modeling. A stepwise technique was used, with testing from the lowest to the highest *p* value. The final model was built with the following assumptions: 1) no change in ORs > 10%; 2) improvement in accuracy by 95% CI; 3) total degrees of freedom allowed for each outcome variable; and 4) quality of the final model, determined by the Hosmer–Lemeshow test. Food covariates were considered adjustments, with control for the confounding variables of sex and age; alcohol consumption in the oral cavity and hypopharyngeal outcomes. For the outcome of oropharyngeal cancer, the interaction between smoking and alcohol consumption was examined, given the change in magnitude of the event. For hypopharyngeal cancer, modeling was performed separately for sociodemographic/lifestyle and food variables because of the small number of incident cases (*n* = 43).

A significance level of 5% (*p* < 0.05) was used. The data were analyzed using IBM SPSS Statistics software version 23.0.

## Results

We recruited 1825 cases and controls initially, of which were excluded by 82 had unspecified HNC, one individual was excluded for being <18 years old, and two for having had received prior treatment for HNC, resulting in a final sample of 1740 subjects (847 cases and 893 controls), including 398 cases of oral cavity cancer, 249 cases of oropharyngeal cancer, 157 cases of laryngeal cancer, and 43 cases of hypopharyngeal cancer.

For all cancers studied, cases and controls differed significantly in terms of educational level, BMI, smoking, alcohol consumption, and oral hygiene; low educational levels, low weight, smoking, alcohol consumption, and poor oral hygiene were more prevalent among cases. A difference in race/skin color between cases and controls was identified only for the oral cavity (Table 1).

**Table 1:** Sociodemographic and lifestyle characteristics of cases with head and neck squamous cell carcinoma (*n* = 847) and healthy controls (*n* = 893).

Variables	Oral cavity				Oropharynx				Larynx				Hypopharynx			
	Total (1194)	Case (398)	Control (796)	p	Total (747)	Case (249)	Control (498)	p	Total (471)	Case (157)	Control (314)	p	Total (172)	Case (43)	Control (129)	p
	n (%)	n (%)	n (%)		n (%)	n (%)	n (%)		n (%)	n (%)	n (%)		n (%)	n (%)	n (%)	
**Sex**																
Male	923 (77.3)	299 (75.1)	624 (78.4)	0.204	663 (88.0)	221 (88.8)	442 (88.8)	0.999	423 (89.9)	141 (89.8)	282 (89.8)	0.999	164 (95.3)	41 (95.3)	123 (95.3)	0.999
Female	271 (22.7)	99 (24.9)	172 (21.6)		84 (11.2)	28 (11.2)	56 (11.2)		48 (10.2)	16 (10.2)	32 (10.2)		8 (4.7)	2 (4.7)	6 (4.7)	
**Age range**																
< 60 years	600 (50.3)	187 (47.0)	413 (51.9)	0.110	471 (63.1)	157 (63.1)	314 (63.1)	0.999	198 (42.0)	66 (42.0)	132 (42.0)	0.999	96 (55.8)	24 (55.8)	72 (55.8)	0.999
≥ 60 years	594 (49.7)	211 (53.0)	383 (48.1)		276 (36.9)	92 (36.9)	184 (36.9)		273 (58.0)	91 (58.0)	182 (58.0)		76 (44.2)	19 (44.2)	57 (44.2)	
**Schooling**																
Illiterate	106 (8.9)	60 (15.2)	46 (5.8)	**<0.001**	61 (8.2)	31 (12.6)	30 (6.0)	**<0.001**	43 (9.1)	27 (17.2)	16 (5.1)	**<0.001**	13 (7.6)	10 (23.3)	3 (2.3)	**<0.001**
Elementary School	698 (58.6)	241 (61.0)	457 (57.4)		438 (58.9)	167 (67.9)	271 (54.4)		274 (58.2)	97 (61.8)	177 (56.4)		100 (58.1)	24 (55.8)	76 (58.9)	
High school	264 (22.2)	55 (13.9)	209 (26.3)		169 (22.7)	28 (11.4)	141 (28.3)		110 (23.4)	23 (14.6)	87 (27.7)		45 (26.2)	8 (18.6)	37 (28.7)	
Higher education	123 (10.3)	39 (9.9)	84 (10.6)		76 (10.2)	20 (8.1)	56 (11.2)		44 (9.3)	10 (6.4)	34 (10.8)		14 (8.1)	1 (2.3)	13 (10.1)	
**Race/skin color**																
White	598 (50.6)	219 (56.2)	379 (47.9)	**0.007**	377 (50.7)	133 (54.1)	244 (49.1)	0.202	244 (51.9)	89 (57.1)	155 (49.4)	0.116	87 (50.6)	18 (41.9)	69 (53.5)	0.187
Non-white	584 (49.4)	171 (43.8)	413 (52.1)		366 (49.3)	113 (45.9)	253 (50.9)		226 (48.1)	67 (42.9)	159 (50.6)		85 (49.4)	25 (58.1)	60 (46.5)	
**BMI (Kg/m**^**2**^**)**																
Normal	468 (39.4)	168 (42.6)	300 (37.8)	**<0.001**	316 (42.6)	120 (49.0)	196 (39.5)	**<0.001**	186 (39.5)	65 (41.4)	121 (38.5)	**<0.001**	75 (43.6)	25 (58.1)	50 (38.8)	**<0.001**
Low weight	193 (16.3)	117 (29.7)	76 (9.6)	** **	108 (14.6)	77 (31.4)	31 (6.3)		83 (17.6)	53 (33.8)	30 (9.6)		22 (12.8)	14 (32.6)	8 (6.2)	
Overweight	318 (26.8)	77 (19.5)	241 (30.4)	** **	185 (25.0)	38 (15.5)	147 (29.6)		125 (26.5)	23 (14.6)	102 (32.5)		43 (25.0)	4 (9.3)	39 (30.2)	
Obese	208 (17.5)	32 (8.1)	176 (22.2)	** **	132 (17.8)	10 (4.1)	122 (24.6)		77 (16.3)	16 (10.2)	61 (19.4)		32 (18.6)	0 (0.0)	32 (24.8)	
**Oral hygiene**																
Good	435 (37.5)	98 (25.5)	337 (43.4)	**<0.001**	263 (36.2)	45 (18.8)	218 (44.9)	**<0.001**	162 (35.3)	39 (25.8)	123 (39.9)	**0.002**	72 (43.9)	8 (20.0)	64 (51.6)	**<0.001**
Reasonable	486 (41.9)	144 (37.4)	342 (44.1)	** **	306 (42.1)	104 (43.3)	202 (41.6)		203 (44.2)	69 (45.7)	134 (43.5)		66 (40.2)	21 (52.5)	45 (36.3)	
Bad	240 (20.7)	143 (37.1)	97 (12.5)	** **	157 (21.6)	91 (37.9)	66 (13.6)		94 (20.5)	43 (28.5)	51 (16.6)		26 (15.9)	11 (27.5)	15 (12.1)	
**Smoker**																
Never smoked	463 (38.9)	75 (19.1)	388 (48.7)	**<0.001**	257 (34.5)	18 (7.3)	239 (48.0)	**<0.001**	156 (33.1)	12 (7.6)	144 (45.9)	**<0.001**	68 (39.5)	5 (11.6)	63 (48.8)	**<0.001**
Former smoker	416 (35.0)	107 (27.2)	309 (38.8)		265 (35.6)	74 (30.1)	191 (38.4)		199 (42.3)	70 (44.6)	129 (41.1)		59 (34.3)	11 (25.6)	48 (37.2)	
Current smoker	310 (26.1)	211 (53.7)	99 (12.4)		222 (29.8)	154 (62.6)	68 (13.7)		116 (24.6)	75 (47.8)	41 (13.1)		45 (26.2)	27 (62.8)	18 (14.0)	
**Alcohol consumption**																
Never drank	377 (31.7)	78 (19.8)	299 (37.6)	**<0.001**	180 (24.3)	17 (6.9)	163 (32.8)	**<0.001**	137 (29.1)	20 (12.7)	117 (37.3)	**<0.001**	39 (22.7)	3 (7.0)	36 (27.9)	**0.003**
Former drinker	386 (32.5)	138 (35.1)	248 (31.2)		270 (36.4)	112 (45.7)	158 (31.8)		184 (39.1)	80 (51.0)	104 (33.1)		68 (39.5)	25 (58.1)	43 (33.3)	
Current drinker	425 (35.8)	177 (45.0)	248 (31.2)		292 (39.4)	116 (47.3)	176 (35.4)		150 (31.8)	57 (36.3)	93 (29.6)		65 (37.8)	15 (34.9)	50 (38.8)	

Chi-squared test. Missing values for oral cavity cancer: education = 3, race = 12, BMI = 7, oral hygiene = 33, smoking = 5, alcohol consumption = 6. Missing values for oropharyngeal cancer: education = 3, race = 4, BMI = 6, oral hygiene = 21, smoking = 3, alcohol consumption = 5. Missing values for laryngeal cancer: race = 1, oral hygiene = 12.

The consumption of minimally processed foods was associated inversely with cancer of the oral cavity, larynx, pharynx, and hypopharynx (Table 2). The consumption of natural juice and citrus fruits was not associated with hypopharyngeal cancer, and the consumption of rice and beans was not associated with any HNC cancer.

**Table 2:** Consumption of minimally processed foods by patients with oral cavity, oropharyngeal, laryngeal, and hypopharyngeal cancers (*n* = 847) and healthy controls (*n* = 893).

Variables	Oral cavity				Oropharynx				Larynx				Hypopharynx			
	Total (1194)	Case (398)	Control (796)	p	Total (747)	Case (249)	Control (498)	p	Total (471)	Case (157)	Control (314)	p	Total (172)	Case (43)	Control (129)	p
	n (%)	n (%)	n (%)		n (%)	n (%)	n (%)		n (%)	n (%)	n (%)		n (%)	n (%)	n (%)	
**Vegetables (except potatoes)**																
Never or <1 time/month	36 (3.0)	29 (7.4)	7 (0.9)	**<0.001**	17 (2.3)	10 (4.1)	7 (1.4)	**0.013**	24 (5.1)	20 (12.7)	4 (1.3)	**<0.001**	12 (7.0)	11 (25.6)	1 (0.8)	**<0.001**
1 to 3 times/month	73 (6.1)	37 (9.4)	36 (4.5)		52 (7.0)	23 (9.4)	29 (5.8)		25 (5.3)	11 (7.0)	14 (4.5)		7 (4.1)	1 (2.3)	6 (4.7)	
1 to 2 times/week	287 (24.1)	109 (27.7)	178 (22.4)		188 (25.3)	70 (28.6)	118 (23.7)		112 (23.8)	47 (29.9)	65 (20.7)		39 (22.7)	11 (25.6)	28 (21.7)	
On most days but not every day	371 (31.2)	101 (25.6)	270 (34.0)		224 (30.2)	65 (26.5)	159 (32.0)		139 (29.5)	41 (26.1)	98 (31.2)		51 (29.7)	7 (16.3)	44 (34.1)	
Everyday	422 (35.5)	118 (29.9)	304 (38.2)		261 (35.2)	77 (31.4)	184 (37.0)		171 (36.3)	38 (24.2)	133 (42.4)		63 (36.6)	13 (30.2)	50 (38.8)	
**Raw greens and vegetables**																
Never or <1 time/month	85 (7.1)	52 (13.2)	33 (4.1)	**<0.001**	46 (6.2)	23 (9.4)	23 (4.6)	**<0.001**	39 (8.3)	26 (16.6)	13 (4.1)	**<0.001**	18 (10.5)	12 (27.9)	6 (4.7)	**<0.001**
1 to 3 times/month	91 (7.6)	45 (11.4)	46 (5.8)		59 (7.9)	26 (10.6)	33 (6.6)		39 (8.3)	22 (14.0)	17 (5.4)		11 (6.4)	4 (9.3)	7 (5.4)	
1 to 2 times/week	362 (30.4)	122 (31.0)	240 (30.2)		248 (33.4)	95 (38.8)	153 (30.7)		146 (31.0)	57 (36.3)	89 (28.3)		50 (29.1)	9 (20.9)	41 (31.8)	
On most days but not every day	349 (29.3)	99 (25.1)	250 (31.4)		206 (27.7)	57 (23.3)	149 (29.9)		130 (27.6)	27 (17.2)	103 (32.8)		49 (28.5)	11 (25.6)	38 (29.5)	
Everyday	303 (25.5)	76 (19.3)	227 (28.5)		184 (24.8)	44 (18.0)	140 (28.1)		117 (24.8)	25 (15.9)	92 (29.3)		44 (25.6)	7 (16.3)	37 (28.7)	
**Broccoli, cabbage, collard greens**															
Never or <1 time/month	119 (10.0)	61 (15.5)	58 (7.3)	**<0.001**	76 (10.2)	35 (14.3)	41 (8.2)	**0.001**	54 (11.5)	36 (22.9)	18 (5.7)	**<0.001**	22 (12.8)	15 (34.9)	7 (5.4)	**<0.001**
1 to 3 times/month	150 (12.6)	60 (15.2)	90 (11.3)		94 (12.7)	41 (16.7)	53 (10.6)		62 (13.2)	30 (19.1)	32 (10.2)		24 (14.0)	7 (16.3)	17 (13.2)	
1 to 2 times/week	483 (40.6)	161 (40.9)	322 (40.5)		317 (42.7)	104 (42.4)	213 (42.8)		181 (38.4)	63 (40.1)	118 (37.6)		67 (39.0)	10 (23.3)	57 (44.2)	
On most days but not every day	285 (23.9)	81 (20.6)	204 (25.6)		159 (21.4)	43 (17.6)	116 (23.3)		113 (24.0)	20 (12.7)	93 (29.6)		38 (22.1)	8 (18.6)	30 (23.3)	
Everyday	153 (12.9)	31 (7.9)	122 (15.3)		97 (13.1)	22 (9.0)	75 (15.1)		61 (13.0)	8 (5.1)	53 (16.9)		21 (12.2)	3 (7.0)	18 (14.0)	
**Carrots**																
Never or <1 time/month	181 (15.2)	94 (23.9)	87 (10.9)	**<0.001**	109 (14.7)	47 (19.2)	62 (12.4)	**<0.001**	83 (17.6)	48 (30.6)	35 (11.1)	**<0.001**	33 (19.2)	20 (46.5)	13 (10.1)	**<0.001**
1 to 3 times/month	172 (14.5)	66 (16.8)	106 (13.3)		127 (17.1)	52 (21.2)	75 (15.1)		67 (14.2)	25 (15.9)	42 (13.4)		28 (16.3)	8 (18.6)	20 (15.5)	
1 to 2 times/week	510 (42.9)	134 (34.0)	376 (47.2)		319 (42.9)	82 (33.5)	237 (47.6)		200 (42.5)	54 (34.4)	146 (46.5)		65 (37.8)	5 (11.6)	60 (46.5)	
On most days but not every day	222 (18.7)	70 (17.8)	152 (19.1)		129 (17.4)	38 (15.5)	91 (18.3)		82 (17.4)	21 (13.4)	61 (19.4)		27 (15.7)	5 (11.6)	22 (17.1)	
Everyday	105 (8.8)	30 (7.6)	75 (9.4)		59 (7.9)	26 (10.6)	33 (6.6)		39 (8.3)	9 (5.7)	30 (9.6)		19 (11.0)	5 (11.6)	14 (10.9)	
**Fresh fruit**																
Never or <1 time/month	75 (6.3)	48 (12.2)	27 (3.4)	**<0.001**	56 (7.5)	38 (15.6)	18 (3.6)	**<0.001**	41 (8.7)	31 (19.7)	10 (3.2)	**<0.001**	15 (8.7)	10 (23.3)	5 (3.9)	**<0.001**
1 to 3 times/month	134 (11.3)	74 (18.8)	60 (7.5)		74 (10.0)	28 (11.5)	46 (9.2)		38 (8.1)	20 (12.7)	18 (5.7)		18 (10.5)	7 (16.3)	11 (8.5)	
1 to 2 times/week	311 (26.1)	111 (28.2)	200 (25.1)		208 (28.0)	67 (27.5)	141 (28.3)		113 (24.0)	41 (26.1)	72 (22.9)		39 (22.7)	10 (23.3)	29 (22.5)	
On most days but not every day	267 (22.4)	73 (18.5)	194 (24.4)		167 (22.5)	55 (22.5)	112 (22.5)		107 (22.7)	34 (21.7)	73 (23.2)		36 (20.9)	5 (11.6)	31 (24.0)	
Everyday	403 (33.9)	88 (22.3)	315 (39.6)		237 (31.9)	56 (23.0)	181 (36.3)		172 (36.5)	31 (19.7)	141 (44.9)		64 (37.2)	11 (25.6)	53 (41.1)	
**Fresh fruit juices**																
Never or <1 time/month	261 (22.0)	102 (26.0)	159 (20.0)	**0.032**	175 (23.6)	75 (30.6)	100 (20.1)	**0.002**	122 (25.9)	64 (40.8)	58 (18.5)	**<0.001**	41 (23.8)	11 (25.6)	30 (23.3)	0.395
1 to 3 times/month	191 (16.1)	65 (16.5)	126 (15.8)		111 (14.9)	28 (11.4)	83 (16.7)		82 (17.4)	27 (17.2)	55 (17.5)		34 (19.8)	8 (18.6)	26 (20.2)	
1 to 2 times/week	379 (31.9)	111 (28.2)	268 (33.7)		237 (31.9)	64 (26.1)	173 (34.7)		129 (27.4)	32 (20.4)	97 (30.9)		45 (26.2)	8 (18.6)	37 (28.7)	
On most days but not every day	198 (16.7)	72 (18.3)	126 (15.8)		110 (14.8)	44 (18.0)	66 (13.3)		68 (14.4)	18 (11.5)	50 (15.9)		23 (13.4)	5 (11.6)	18 (14.0)	
Everyday	160 (13.5)	43 (10.9)	117 (14.7)		110 (14.8)	34 (13.9)	76 (15.3)		70 (14.9)	16 (10.2)	54 (17.2)		29 (16.9)	11 (25.6)	18 (14.0)	
**Apples or pears**																
Never or <1 time/month	229 (19.2)	135 (34.3)	94 (11.8)	**<0.001**	148 (19.9)	85 (34.7)	63 (12.7)	**<0.001**	96 (20.4)	66 (42.0)	30 (9.6)	**<0.001**	41 (23.8)	20 (46.5)	21 (16.3)	**0.002**
1 to 3 times/month	236 (19.8)	79 (20.1)	157 (19.7)		156 (21.0)	49 (20.0)	107 (21.5)		94 (20.0)	31 (19.7)	63 (20.1)		42 (24.4)	8 (18.6)	34 (26.4)	
1 to 2 times/week	417 (35.0)	116 (29.4)	301 (37.8)		260 (35.0)	73 (29.8)	187 (37.6)		155 (32.9)	39 (24.8)	116 (36.9)		44 (25.6)	6 (14.0)	38 (29.5)	
On most days but not every day	195 (16.4)	44 (11.2)	151 (19.0)		115 (15.5)	27 (11.0)	88 (17.7)		66 (14.0)	11 (7.0)	55 (17.5)		27 (15.7)	5 (11.6)	22 (17.1)	
Everyday	113 (9.5)	20 (5.1)	93 (11.7)		64 (8.6)	11 (4.5)	53 (10.6)		60 (12.7)	10 (6.4)	50 (15.9)		18 (10.5)	4 (9.3)	14 (10.9)	
**Citrus fruit (oranges, lemons, tangerines)**																
Never or <1 time/month	133 (11.2)	82 (20.8)	51 (6.4)	**<0.001**	78 (10.5)	45 (18.4)	33 (6.6)	**<0.001**	49 (10.4)	29 (18.5)	20 (6.4)	**<0.001**	17 (9.9)	7 (16.3)	10 (7.8)	0.121
1 to 3 times/month	172 (14.5)	79 (20.1)	93 (11.7)		100 (13.5)	34 (13.9)	66 (13.3)		65 (13.8)	27 (17.2)	38 (12.1)		29 (16.9)	11 (25.6)	18 (14.0)	
1 to 2 times/week	375 (31.5)	123 (31.2)	252 (31.7)		253 (34.1)	87 (35.5)	166 (33.3)		120 (25.5)	48 (30.6)	72 (22.9)		42 (24.4)	9 (20.9)	33 (25.6)	
On most days but not every day	245 (20.6)	57 (14.5)	188 (23.6)		150 (20.2)	38 (15.5)	112 (22.5)		97 (20.6)	23 (14.6)	74 (23.6)		35 (20.3)	8 (18.6)	27 (20.9)	
Everyday	265 (22.3)	53 (13.5)	212 (26.6)		162 (21.8)	41 (16.7)	121 (24.3)		140 (29.7)	30 (19.1)	110 (35.0)		49 (28.5)	8 (18.6)	41 (31.8)	
**Fresh tomatoes**																
Never or <1 time/month	101 (8.5)	66 (16.8)	35 (4.4)	**<0.001**	42 (5.7)	22 (9.0)	20 (4.0)	**0.023**	44 (9.3)	27 (17.2)	17 (5.4)	**<0.001**	20 (11.6)	11 (25.6)	9 (7.0)	**0.012**
1 to 3 times/month	87 (7.3)	40 (10.2)	47 (5.9)		56 (7.5)	21 (8.6)	35 (7.0)		32 (6.8)	12 (7.6)	20 (6.4)		4 (2.3)	0 (0.0)	4 (3.1)	
1 to 2 times/week	310 (26.1)	109 (27.7)	201 (25.3)		209 (28.1)	74 (30.2)	135 (27.1)		111 (23.6)	43 (27.4)	68 (21.7)		39 (22.7)	10 (23.3)	29 (22.5)	
On most days but not every day	347 (29.2)	102 (25.9)	245 (30.8)		210 (28.3)	58 (23.7)	152 (30.5)		130 (27.6)	37 (23.6)	93 (29.6)		46 (26.7)	11 (25.6)	35 (27.1)	
Everyday	345 (29.0)	77 (19.5)	268 (33.7)		226 (30.4)	70 (28.6)	156 (31.3)		154 (32.7)	38 (24.2)	116 (36.9)		63 (36.6)	11 (25.6)	52 (40.3)	
**Bananas**																
Never or <1 time/month	77 (6.5)	47 (11.9)	30 (3.8)	**<0.001**	57 (7.7)	37 (15.2)	20 (4.0)	**<0.001**	31 (6.6)	18 (11.5)	13 (4.1)	**<0.001**	10 (5.8)	6 (14.0)	4 (3.1)	**0.044**
1 to 3 times/month	94 (7.9)	53 (13.5)	41 (5.2)		45 (6.1)	17 (7.0)	28 (5.6)		35 (7.4)	17 (10.8)	18 (5.7)		12 (7.0)	3 (7.0)	9 (7.0)	
1 to 2 times/week	295 (24.8)	112 (28.4)	183 (23.0)		192 (25.9)	70 (28.7)	122 (24.5)		96 (20.4)	47 (29.9)	49 (15.6)		26 (15.1)	9 (20.9)	17 (13.2)	
On most days but not every day	284 (23.9)	94 (23.9)	190 (23.9)		169 (22.8)	49 (20.1)	120 (24.1)		114 (24.2)	33 (21.0)	81 (25.8)		47 (27.3)	11 (25.6)	36 (27.9)	
Everyday	440 (37.0)	88 (22.3)	352 (44.2)		279 (37.6)	71 (29.1)	208 (41.8)		195 (41.4)	42 (26.8)	153 (48.7)		77 (44.8)	14 (32.6)	63 (48.8)	
**Rice and beans**																
Do not consume rice and beans or consume little	64 (5.4)	27 (6.9)	37 (4.6)	0.086	44 (5.9)	19 (7.8)	25 (5.0)	0.427	20 (4.2)	8 (5.1)	12 (3.8)	0.398	5 (2.9)	1 (2.3)	4 (3.1)	0.600
Consume rice and consume little beans	69 (5.8)	17 (4.3)	52 (6.5)		27 (3.6)	7 (2.9)	20 (4.0)		24 (5.1)	11 (7.0)	13 (4.1)		8 (4.7)	1 (2.3)	7 (5.4)	
Consume little rice and consume more beans	9 (0.8)	1 (3)	8 (1.0)		7 (0.9)	2 (0.8)	5 (1.0)		6 (1.3)	1 (0.6)	5 (1.6)		3 (1.7)	0 (0.0)	3 (2.3)	
Consume rice and beans almost daily	1048 (88.1)	349 (88.6)	699 (87.8)		665 (89.5)	217 (88.6)	448 (90.0)		421 (89.4)	137 (87.3)	284 (90.4)		156 (90.7)	41 (95.3)	115 (89.1)	

Chi-squared test. Missing values for oral cavity cancer: vegetables and fresh fruit juice = 5, other food = 4. Missing values for oropharyngeal cancer: vegetables, fresh fruit juice, and bananas = 5; other food = 4.

### Oral cavity cancer

Past and current smoking increased the risk of oral cavity cancer occurrence, with ORs of 1.54 (95% CI 1.01–2.34, *p* = 0.043) and 7.17 (95% CI 4.42–11.12, *p* < 0.001), respectively, compared with individuals who never smoked. Participants with poor/bad oral hygiene were twice as likely to have oral cavity cancer as were those with good hygiene (OR 2.14, 95% CI 1.34–3.42, *p* = 0.002).

Compared with illiterate participants, those who had completed primary education were 60% less likely to have oral cavity cancer (OR 0.40, 95% CI 0.23–0.71, *p* = 0.002), and those with high-school educations were 75% less likely to have this cancer type (OR 0.25, 95% CI 0.12–0.46, *p* < 0.001). Subjects who self-reported their race/skin color as non-white had a 58% lesser chance of having oral cavity cancer than were those who identified themselves as white (OR 0.42, 95% CI 0.30–0.59, *p* < 0.001). Individuals with low weights had a 3.78 times greater chance of having oral cavity cancer than did those with excess weight (OR 3.78, 95% CI 2.40–5.96, *p* < 0.001).

Greater consumption of minimally processed foods was associated with a reduced chance of having oral cavity cancer. The consumption of apples and pears on most days reduced this chance by 59% (OR 0.41, 95% CI 0.22–0.76, *p* = 0.004), and everyday consumption reduced the chance by 66% (OR 0.34, 95% CI 0.17–0.71, *p* = 0.004). Citrus fruit consumption reduced the risk by up to 66% (OR 0.34, 95% CI 0.17–0.66, *p* = 0.002). The consumption of fresh tomatoes every day reduced the chance of having oral cavity cancer by 72% (OR 0.28, 95% CI 0.14–0.56, *p* < 0.001; Table 3).

**Table 3:** Associations of lifestyle and dietary habits with oral cavity cancer, as determined by multiple binary logistic regression.

Variables	N		OR_adjusted_	95% CI		p
	Case	Control		Lower	Upper	
**Smoker**						
Never smoked	74	374	1			
Former smoker	101	297	1.54	1.01	2.34	**0.043**
Current smoker	200	97	7.17	4.52	11.37	**<0.001**
**Schooling**						
Illiterate	57	43	1			
Elementary school	229	439	0.40	0.23	0.71	**0.002**
High school	52	203	0.25	0.13	0.49	**<0.001**
Higher education	37	83	0.58	0.38	1.22	0.152
**Race/skin color**						
White	214	370	1			
Non-white	161	398	0.42	0.30	0.59	**<0.001**
**Oral hygiene**						
Good	96	331	1			
Reasonable	141	341	0.94	0.64	1.37	0.732
Bad	138	96	2.14	1.34	3.42	**0.002**
**BMI (Kg/m**^**2**^**)**						
Normal	156	290	1			
Low weight	112	73	3.78	2.40	5.96	**<0.001**
Overweight/obese	107	405	1.35	0.95	1.92	0.097
**Apples or pears**						
Never or <1 time/month	103	87	1			
1 to 3 times/month	74	150	0.58	0.35	0.98	**0.043**
1 to 2 times/week	109	292	0.51	0.31	0.82	**0.006**
On most days but not every day	42	147	0.41	0.22	0.76	**0.004**
Everyday	20	92	0.34	0.17	0.66	**0.004**
**Citrus fruit (oranges, lemons, tangerines)**						
Never or <1 time/month	78	48	1			
1 to 3 times/month	77	87	0.84	0.44	1.59	0.582
1 to 2 times/week	117	243	0.52	0.29	0.93	**0.026**
On most days but not every day	52	182	0.35	0.18	0.67	**0.002**
Everyday	51	208	0.34	0.17	0.66	**0.002**
**Fresh tomatoes**						
Never or <1 time/month	62	35	1			
1 to 3 times/month	39	43	0.65	0.29	1.45	0.291
1 to 2 times/week	105	193	0.43	0.22	0.83	**0.012**
On most days but not every day	94	234	0.32	0.16	0.62	**0.001**
Everyday	75	263	0.28	0.14	0.56	**<0.001**

The model was adjusted for the consumption of “vegetables (except potatoes),” "natural fruit juice," "carrots," "raw greens and vegetables," "rice and beans," and "alcohol." Hosmer–Lemeshow value = 0.706. OR, odds ratio; CI, confidence interval; BMI, body mass index.

### Oropharyngeal cancer

Current smoking and alcohol consumption increased the chance of having oropharyngeal cancer by 18 times compared with never smoking or drinking (OR 18.26, 95% CI 8.19–40.73, *p* < 0.001). Compared with individuals who never smoked or drank, ORs for oropharyngeal cancer were 4.79 (95% CI 2.24–10.22, *p* < 0.0001) among individuals who formerly smoked or drank, 3.07 (95% CI 1.34–7.03, *p* = 0.008) among those who currently smoked but never drank, and 16.22 (95% CI 6.93–37.95, *p* < 0.001) among former drinkers who currently smoked.

Individuals with poor/bad oral hygiene were twice as likely to have oropharyngeal cancer as were those with good oral hygiene (OR 2.1, 95% CI 1.13–3.89, *p* = 0.022). Individuals of non-white race/skin color had half the chance of having oropharyngeal cancer as did those who identified as white (OR 0.50, 95% CI 0.32–0.77, *p* = 0.002). Low-weight individuals were 4.11 times more likely (95% CI 2.19–7.72, *p* < 0.001) and overweight subjects were 52% less likely (OR 0.48, 95% CI 0.30–0.77, *p* = 0.002) to have oropharyngeal cancer than were those with normal BMIs.

The consumption of bananas every day reduced the odds of having oropharyngeal cancer by 77% compared with never consuming bananas (OR 0.23, 95% CI 0.09–0.55, *p* = 0.001; Table 4).

**Table 4:** Associations of lifestyle and dietary habits with oropharyngeal cancer, as determined by multiple binary logistic regression.

Variables	n		OR_adjusted_	95% CI		p
	Case	Control		Lower	Upper	
**Smoking and alcohol interaction**						
Never smoked and never drank	12	110	1			
Never smoked, but drank in the past/Never drank, but smoked in the past	9	158	0.56	0.22	1.46	0.236
Smoked and drank in the past	45	87	4.79	2.24	10.22	**<0.001**
Smokes currently, but never drank	22	72	3.07	1.34	7.03	**0.008**
Currently smokes and drank in the past	58	22	16.22	6.93	37.95	**<0.001**
Smokes and Drinks Currently	86	33	18.26	8.19	40.73	**<0.001**
**Oral hygiene**						
Good	45	215	1			
Reasonable	100	202	1.51	0.90	2.53	0.118
Bad	87	65	2.10	1.13	3.89	**0.019**
**Race/skin color**						
White	126	238	1			
Non-white	106	244	0.50	0.32	0.77	**0.002**
**BMI (Kg/m**^**2**^**)**						
Normal	112	189	1			
Low weight	74	30	4.11	2.19	7.72	**<0.001**
Overweight/obese	46	263	0.48	0.30	0.77	**0.002**
**Bananas**						
Never or <1 time/month	34	20	1			
1 to 3 times/month	15	28	0.49	0.16	1.49	0.208
1 to 2 times/week	65	114	0.52	0.22	1.22	0.133
On most days but not every day	48	115	0.31	0.13	0.75	**0.009**
Everyday	70	205	0.23	0.09	0.55	**0.001**

The model was adjusted for the consumption of "citrus fruits (oranges, lemons, and tangerines)" and "carrots." Hosmer–Lemeshow value = 0.918. BMI: Body Mass Index. OR, odds ratio; CI, confidence interval; BMI, body mass index.

### Laryngeal cancer

Former and current smoking increased the odds of having laryngeal cancer by 3.93 (95% CI 1.88–8.24, *p* < 0.001) and 8.18 (95% CI 3.61–18.55, *p* < 0.001) times, respectively, compared with never smoking. Alcohol consumption in the past increased the chance of having this cancer by 2.23 times (95% CI 1.10–4.54, *p* = 0.027) relative to never drinking. Compared with normal-weight subjects, those with low weights had an approximately three times greater chance (OR 2.89, 95% CI 1.46–5.71, *p* < 0.001), and overweight subjects had a 54% lesser chance (95% CI 0.32–0.97, *p* = 0.038) of having laryngeal cancer.

Increased consumption of apples and pears reduced the chance of having laryngeal cancer; consumption on most days or every day reduced this chance by 74% (OR 0.26, 95% CI 0.10–0.66, *p* = 0.005) compared with rare or no consumption. Consumption of broccoli, cabbage, and/or collard greens on most days and daily reduced the chance of having laryngeal cancer by 79% (OR 0.21, 95% CI 0.08–0.57, *p* = 0.002) and 80% (OR 0.20, 95% CI 0.06–0.66, *p* = 0.008), respectively (Table 5).

**Table 5:** Associations of lifestyle and dietary habits with laryngeal cancer, as determined by multiple binary logistic regression.

Variables	n		OR_adjusted_	95% CI		p
	Case	Control		Lower	Upper	
**Smoker**						
Never smoked	‘10	141	1			
Former smoker	70	126	3.93	1.88	8.24	**<0.001**
Current smoker	71	41	8.18	3.61	18.55	**<0.001**
**Alcohol drinker**						
Never drank	19	116	1			
Former drinker	78	100	2.23	1.10	4.54	0.027
Current drinker	54	92	2.10	1.00	4.38	0.050
**BMI (Kg/m**^**2**^**)**						
Normal	65	118	1			
Low weight	49	30	2.89	1.46	5.71	**<0.001**
Overweight/obese	37	160	0.55	0.32	0.97	**0.038**
**Apples or pears**						
Never or <1 time/month	62	26	1			
1 to 3 times/month	31	62	0.40	0.19	0.84	**0.016**
1 to 2 times/week	39	116	0.37	0.19	0.75	**0.005**
On most days but not every day	9	55	0.26	0.10	0.66	**0.005**
Everyday	10	49	0.26	0.10	0.68	**0.006**
**Broccoli, cabbage, collard greens**						
Never or <1 time/month	36	18	1			
1 to 3 times/month	28	31	0.86	0.32	2.30	0.756
1 to 2 times/week	62	116	0.56	0.24	1.36	0.201
On most days but not every day	18	91	0.21	0.08	0.57	**0.002**
Everyday	7	52	0.20	0.06	0.66	**0.008**

The model was adjusted for the consumption of "carrots" and for education. Hosmer–Lemeshow value = 0.982. OR, odds ratio; CI, confidence interval; BMI, body mass index.

### Hypopharyngeal cancer

Smoking increased the odds of having hypopharyngeal cancer by almost nine times compared with never smoking (OR 8.74, 95% CI 2.32–32.91, *p* = 0.001). Primary and secondary education levels reduced the chance of having this type of tumor by 91% relative to illiteracy (OR 0.09, 95% CI 0.01–0.67, *p* = 0.019 and OR 0.09, 95% CI 0.01–0.60, *p* = 0.013, respectively). Subjects with low weight had 34.87 times the odds (95% CI 6.52–186.66, *p* < 0.001), and overweight subjects had a 91% lesser chance (OR 0.09, 95% CI 0.02–0.35, *p* = 0.001) of having hypopharyngeal cancer than did those with normal BMIs.

Consumption of broccoli, cabbage, and/or collard greens 1–2 times/week decreased the chance of having hypopharyngeal cancer by 69% (OR 0.31, 95% CI 0.11–0.87, *p* = 0.026) compared with consumption of these items fewer than 3 times/month. Likewise, carrot consumption decreased the odds of having this tumor type by 86% (OR 0.14, 95% CI 0.04–0.44, *p* = 0.001). Consumption of fresh fruits almost every day or more reduced the chance of having hypopharyngeal cancer by 73% (OR 0.27, 95% CI 0.08–0.96, *p* = 0.0.042; Table 6).

**Table 6:** Associations of lifestyle and dietary habits with hypopharyngeal cancer, as determined by multiple binary logistic regression.

**Model 1- Demographic and lifestyle characteristics**						
**Variables**	**n**		**OR**_adjusted_*	**95% CI**		**p**
	**Case**	**Control**		**Lower**	**Upper**	
**Smoker**						
Never smoked	5	63	1			
Former smoker	11	48	3.34	0.88	12.72	0.077
Current smoker	27	18	8.74	2.32	32.91	**0.001**
**Education**						
Illiterate	10	3	1			
High school	50	76	0.09	0.01	0.60	**0.013**
≥ Basic education	9	50	0.09	0.01	0.67	**0.019**
**BMI (Kg/m**^**2**^**)**						
Normal	25	50	1			
Low weight	14	8	3.07	0.91	10.38	0.071
Overweight	4	71	0.09	0.02	0.35	**0.001**
**Model 2 –Food characteristics**						
**Variables**	**n**		**OR**_**adjusted**__†_	**95% CI**		**p**
	**Case**	**Control**		**Lower**	**Upper**	
**Broccoli, cabbage, collard greens**						
<3 times/month	22	24	1			
1 to 2 times/week	10	57	0.31	0.11	0.87	**0.026**
Almost every day or more	11	48	0.34	0.11	1.06	0.064
**Carrot**						
<3 times/month	28	33	1			
1 to 2 times/week	5	60	0.14	0.04	0.44	**0.001**
Almost every day or more	10	36	0.51	0.17	1.52	0.228
**Fresh fruit**						
<3 times/month	17	16	1			
1 to 2 times/week	10	29	0.89	0.25	3.14	0.852
Almost every day or more	16	84	0.27	0.08	0.96	**0.042**

*The model was adjusted for alcohol consumption. Hosmer–Lemeshow value = 0.961.

†The model was adjusted for consumption of "apples and pears" and "citrus fruits (oranges, lemons, and tangerines)." Hosmer–Lemeshow value = 0.899. OR, odds ratio; CI, confidence interval; BMI, body mass index.

## Discussion

In this study, we observed that the consumption of minimally processed foods, especially key protective fruits and vegetables (citrus fruits, tomatoes, cruciferous vegetables, apples, pears, and bananas), reduced the chance of having squamous cell carcinoma of the head and neck. Minimally processed foods contribute to the prevention of chronic noncommunicable diseases, as their consumption results in diets with low energy density and low levels of free sugars, unhealthy fats, and salt, as well as large amounts of fiber [22]. Several mechanisms may be involved in the reduction of cancer risk provided by the consumption of these foods, as the nutrients and phytochemicals that they contain may interfere in different stages of carcinogenesis [16,23].

Evidence has accumulated on the prevention of cancer by bioactive components, such as lycopene from tomatoes, isothiocyanates in cruciferous vegetables such as broccoli, and monoterpenes from citrus fruit [16], and is supported by the findings of the present study. An analysis of 805 cases of oral and pharyngeal cancer showed that the dietary consumption of flavonoids reduced the probability of developing such tumors by 50% [12] because flavonoids regulate the expression and action of several microRNAs in different cancers [16,24,25]. Likewise, the protective effect found for apples and pears is due to the presence of quercetin, which induces cell cycle arrest and apoptosis [23]. Lycopene, present in tomatoes, has antioxidant actions, inhibiting the growth of tumor cells in humans and preventing the proinflammatory production of interleukin 8 induced by smoking [26,27], which explain the possible associations found in this study. In a pooled analysis of 10 case-control studies, Leoncini et al. [28] found a 40% reduction in the chance of having cancers of the mouth, pharynx, and larynx in individuals with higher carotenoid consumption.

Cruciferous consumption was associated with a reduction in the risk of laryngeal and hypopharyngeal cancers in this study, probably due to the presence of natural sulfur compounds known as glucosinolates, which are a nutritional source of thiocyanates and isothiocyanates [23,29]. These molecules can block the action of carcinogens and suppress the expression of neoplasia in initiated cancer cells [29], facilitating the detoxification and excretion of carcinogens, protecting against oxidative stress, inhibiting the proliferation of cancer cells, and increasing apoptosis [23]. In addition to the systemic protective effects of phytochemicals, polyphenols may exert local actions during the chewing of food through contact with tissues, inhibiting the proliferation of cancer cells on the surfaces of epithelial cells and thereby preventing cancer of the mouth [30].

Banana consumption was associated with protective effects for oropharyngeal cancer. This fruit contains several bioactive compounds, such as vitamins, phenolic acids, carotenoids, biogenic amines, and phytosterols, which are highly desirable in the diet because they exert antioxidant effects [31,32]. In addition, bananas are widely appreciated in many countries because of their high nutritional value and low cost [33]. Bananas are the most popular fruit in Brazil, being the most consumed food after rice, beans, coffee, bread, salt, and beef [30].

The protective effects of fresh fruits can be explained by the numerous bioactive compounds present in these foods. In addition to attempts to explain the roles of nutrients and phytochemicals in the genesis and prevention of cancer, interest in the study of dietary patterns and their synergistic effects is increasing [7,34–39], as diets rich in anti-inflammatory agents from various dietary sources may help to reduce the risk of cancers of the mouth and pharynx [36,40,41].

Combined food chemoprevention strategies produce "pharmacodynamic synergy," in which the impact of the phytochemical mixture is more prominent than the impacts of isolated phytochemicals [42]. Tseng [43] demonstrated in 2009 that at least 20% of all cancers can be prevented by the consumption of diets rich in vegetables and fruits (>400 g/day), precisely because these types of food contain mixtures of phytochemicals and act synergistically [43,44]. Many experimental and in vitro studies have not produced the same results as studies assessing individuals’ food consumption due to inherent differences between induced laboratory conditions and actual human physiological conditions [37,45].

In an analysis of 14,852 cases of HNC, the International Consortium for the Epidemiology of Head and Neck Cancer (INHANCE) determined that the highest overall intake of fruits, particularly citrus fruits, apples, and pears, led to the lowest risk of this type of cancer (OR 0.52, 95% CI 0.43–0.62, *p* < 0.01). Similarly, individuals with higher vegetable intakes had a lower risk of HNC (OR 0.66, 95% CI 0.49–0.90, *p* = 0.01). In addition, the consumption of green salads, lettuce, and fresh tomatoes more than 7 times per week was associated with a lower risk of having this type of tumor [34]. Another study with HNC and smoking showed that higher frequencies of fruit and vegetable intake were associated inversely with the risk of this cancer in all age groups [7].

Our study revealed no significant difference in the risk of the cancers examined related to the consumption of rice and beans. In contrast, Marchioni et al. [14] reported that beans protected against oral cancer, with a significant tendency for risk reduction with increased consumption, in a population from São Paulo. The lack of association in the present study may be due to the high intake of these foods overall in the study population, as rice and beans remain the basis of Brazilian diets [46].

Other risk factors for HNC identified in this study were similar to those found in the literature. In a French multicenter study, subjects with HNC had lower educational levels and greater tobacco use and alcohol consumption than did controls [47]. Individual studies that contributed to the INHANCE consortium documented strong associations between the duration and intensity of tobacco and alcohol use and the risk of HNC; smoking cessation reduced this risk, whereas the effect of alcohol withdrawal was not clear [11].

Smokers and heavy drinkers have higher levels of inflammation markers [48,49], and a phytochemical-rich diet may be more effective in these individuals [41,48]. Cruciferous extracts induce the actions of cytoprotective enzymes, such as glucorapanin, which promote the detoxification of carcinogenic chemical agents, including benzene, aldehydes, and polycyclic aromatic hydrocarbons found in tobacco smoke [50].

Nutritional status at diagnosis is associated with low weight status. According to Magnano et al. [51], malnutrition is a typical characteristic of patients with HNC because they underwent progressive and involuntary weight loss even before cancer treatment. In our study, 31.1% (n = 261) of all cases had malnutrition at diagnosis of HNC.

A higher level of education has been deemed a protective factor for HNC [52–55]. The association of low education, low income, and non-white race/skin color with HNC development has been related to the roles of these factors as social determinants of health, lifestyle, and behavioral factors, food choices, and/or psychosocial factors [56]. A higher level of education was a determinant of better-quality diets among patients with cancer [54]; in turn, poor oral hygiene has been associated with a higher risk of HNC [57], as found in our study.

Few studies have been conducted in Brazil to investigate the association between diet and HNC [7,14,58–61]. However, scientific evidence for the relationships between HNC in different locations and some types of food and nutrients remains insufficient or inconsistent. Several factors may explain this situation and can be considered to be limitations of this work, including factors inherent to case-control studies, such as memory bias, the consumption of foods and nutrients associated with cancer many years before cancer onset, and patients’ modification of their diets during the initial (prediagnosis) phases of the disease [62]. However, our data on the protective effects of minimally processed plant foods against HNC are supported by the strengths of associations, consistency of results, and biological plausibility of the findings.

In addition, this study is among the first to assess the effects of protective foods in terms of HNC risk according to the Food Rating Approach for the Brazilian Population [18]. It is also among the largest multicenter studies of the cancers examined conducted in Brazil; participating centers were in three Brazilian states. Other studies that have investigated dietary associations with the risk of HNC in Brazil have used data only from São Paulo [7,14,58,60,61] or Rio de Janeiro [59]. Moreover, the analyses in our study were subdivided by four HNC locations, in an effort to determine whether the protective role of minimally processed foods differed among these locations; in contrast, previous studies have focused only on oral and oropharyngeal cancers [14,58,60,61] or oral and pharyngeal cancers [59].

## Conclusions

In multiple analyses adjusted for smoking, alcohol consumption, poor oral hygiene, and low educational level, the consumption of minimally processed foods reduced the chance of HNC development. The consumption of apples and pears was associated with reduced risks of oral cavity and laryngeal cancers; the consumption of citrus fruits and fresh tomatoes was associated with a reduced risk of oral cavity cancer; the consumption of bananas was associated with a reduced risk of oropharynx cancer; the consumption of broccoli, cabbage, and collard greens was associated with reduced risks of laryngeal and hypopharyngeal cancers; and the consumption of carrots and fresh fruits was associated with a reduced risk of hypopharyngeal cancer. The findings of our study support the adoption of preventive measures for HNC that encourage the consumption of minimally processed foods. Incentives may take the form of public education policies and address nutritional status control, oral hygiene, restriction of alcohol consumption, and cessation of tobacco use. The facilitation of access to minimally processed foods through public policies, such as subsidy provision for home and community gardens and free markets, is essential, and will strengthen the development and consolidation of policies that aim to create environments conducive to healthy behavior.

## Supporting information

S1 Table OR, odds ratio; CI, confidence interval; BMI, body mass index. (DOC)
