# Supplementary material for: Consumption of minimally processed foods as protective factors in the genesis of squamous cell carcinoma of the head and neck in Brazil
Source: PLoS One. 2019 Jul 25;14(7):e0220067. doi: 10.1371/journal.pone.0220067 (PMC6657870; doi:10.1371/journal.pone.0220067)
Supplement: S1 Table — (DOC) [file pone.0220067.s001.doc]

**S1 Table** – Univariate binary logistic regression analysis of lifestyle and dietary habits with oral cavity, oropharynx, laryngeal and hipopharynx cancers

| **Variables** | **Oral Cavity** | | | | **Oropharynx** | | | | **Larynx** | | | | **Hipopharynx** | | | |
| --- | --- | --- | --- | --- | --- | --- | --- | --- | --- | --- | --- | --- | --- | --- | --- | --- |
|  | **OR** | **95% CI** | | **p** | **OR** | **95% CI** | | **p** | **OR** | **95% CI** | | **p** | **OR** | **95% CI** | | **p** |
|  |  | Lower | Upper |  |  | Lower | Upper |  |  | Lower | Upper |  |  | Lower | Upper |  |
| **Schooling** |  |  |  |  |  |  |  |  |  |  |  |  |  |  |  |  |
| Illiterate | 1 |  |  |  | 1 |  |  |  | 1 |  |  |  | 1 |  |  |  |
| Elementary school | 0.40 | 0.27 | 0.61 | <0.001 | 0.60 | 0.35 | 1.02 | 0.060 | 0.32 | 0.17 | 0.63 | 0.001 | 0.09 | 0.02 | 0.37 | 0.001 |
| High school | 0.20 | 0.12 | 0.33 | <0.001 | 0.19 | 0.10 | 0.37 | <0.001 | 0.16 | 0.07 | 0.34 | <0.001 | 0.06 | 0.01 | 0.29 | <0.001 |
| Higher education | 0.36 | 0.21 | 0.61 | <0.001 | 0.35 | 0.17 | 0.71 | 0.004 | 0.17 | 0.07 | 0.45 | <0.001 | 0.02 | 0.00 | 0.26 | 0.002 |
| **Race/skin color** |  |  |  |  |  |  |  |  |  |  |  |  |  |  |  |  |
| White | 1 |  |  |  | 1 |  |  |  | 1 |  |  |  | 1 |  |  |  |
| Non-white | 0.72 | 0.56 | 0.91 | 0.007 | 0.82 | 0.60 | 1.11 | 0.202 | 0.73 | 0.50 | 1.08 | 0.117 | 1.60 | 0.79 | 3.21 | 0.188 |
| **BMI (Kg/m²)** |  |  |  |  |  |  |  |  |  |  |  |  |  |  |  |  |
| Eutrophic | 1 |  |  |  | 1 |  |  |  | 1 |  |  |  | 1 |  |  |  |
| Low weight | 2.75 | 1.95 | 3.88 | <0.001 | 4.06 | 2.52 | 6.52 | <0.001 | 3.29 | 1.92 | 5.64 | <0.001 | 3.50 | 1.29 | 9.44 | 0.013 |
| Overweigth | 0.47 | 0.35 | 0.62 | <0.001 | 0.29 | 0.20 | 0.43 | <0.001 | 0.45 | 0.28 | 0.71 | 0.001 | 0.11 | 0.04 | 0.34 | <0.001 |
| **Oral hygiene** |  |  |  |  |  |  |  |  |  |  |  |  |  |  |  |  |
| Good | 1 |  |  |  | 1 |  |  |  | 1 |  |  |  | 1 |  |  |  |
| Reasonable | 1.45 | 1.08 | 1.95 | 0.015 | 2.49 | 1.67 | 3.72 | <0.001 | 1.62 | 1.02 | 2.58 | 0.040 | 3.73 | 1.52 | 9.17 | 0.004 |
| Bad | 5.07 | 3.60 | 7.14 | <0.001 | 6.68 | 4.25 | 10.49 | <0.001 | 2.66 | 1.55 | 4.57 | <0.001 | 5.87 | 2.01 | 17.11 | 0.001 |
| **Smoker** |  |  |  |  |  |  |  |  |  |  |  |  |  |  |  |  |
| Never smoked | 1 |  |  |  | 1 |  |  |  | 1 |  |  |  | 1 |  |  |  |
| Former smoker | 1.79 | 1.29 | 2.49 | 0.001 | 5.14 | 2.97 | 8.91 | <0.001 | 6.51 | 3.38 | 12.56 | <0.001 | 2.89 | 0.94 | 8.87 | 0.064 |
| Current smoker | 11.03 | 7.82 | 15.55 | <0.001 | 30.07 | 17.22 | 52.52 | <0.001 | 21.95 | 10.89 | 44.26 | <0.001 | 18.90 | 6.36 | 56.13 | <0.001 |
| **Alcohol drinker** |  |  |  |  |  |  |  |  |  |  |  |  |  |  |  |  |
| Never drank | 1 |  |  |  | 1 |  |  |  | 1 |  |  |  | 1 |  |  |  |
| Former drinker | 2.13 | 1.54 | 2.95 | <0.001 | 6.80 | 3.90 | 11.84 | <0.001 | 4.50 | 2.58 | 7.85 | <0.001 | 6.98 | 1.95 | 25.01 | 0.003 |
| Current drinker | 2.74 | 2.00 | 3.75 | <0.001 | 6.32 | 3.64 | 10.97 | <0.001 | 3.59 | 2.01 | 6.39 | <0.001 | 3.60 | 0.97 | 13.36 | 0.056 |
| **Vegetables (except potatoes)** |  |  |  |  |  |  |  |  |  |  |  |  |  |  |  |  |
| Never or <1 time/month | 1 |  |  |  | 1 |  |  |  | 1 |  |  |  | 1 |  |  |  |
| 1 to 3 times/month | 0.25 | 0.10 | 0.64 | 0.004 | 0.56 | 0.18 | 1.68 | 0.299 | 0.16 | 0.04 | 0.60 | 0.006 | 0.02 | 0.00 | 0.29 | 0.005 |
| 1 to 2 times/week | 0.15 | 0.06 | 0.35 | 0.000 | 0.42 | 0.15 | 1.14 | 0.088 | 0.14 | 0.05 | 0.45 | 0.001 | 0.04 | 0.00 | 0.31 | 0.003 |
| On most days but not every day | 0.09 | 0.04 | 0.21 | 0.000 | 0.29 | 0.10 | 0.78 | 0.015 | 0.08 | 0.03 | 0.26 | <0.001 | 0.01 | 0.00 | 0.13 | <0.001 |
| Everyday | 0.09 | 0.04 | 0.22 | 0.000 | 0.29 | 0.11 | 0.80 | 0.016 | 0.06 | 0.02 | 0.18 | <0.001 | 0.02 | 0.00 | 0.20 | 0.001 |
| **Raw greens and vegetables** |  |  |  |  |  |  |  |  |  |  |  |  |  |  |  |  |
| Never or <1 time/month | 1 |  |  |  | 1 |  |  |  | 1 |  |  |  | 1 |  |  |  |
| 1 to 3 times/month | 0.62 | 0.34 | 1.13 | 0.119 | 0.79 | 0.36 | 1.71 | 0.546 | 0.65 | 0.26 | 1.62 | 0.353 | 0.29 | 0.06 | 1.38 | 0.118 |
| 1 to 2 times/week | 0.32 | 0.20 | 0.53 | 0.000 | 0.62 | 0.33 | 1.17 | 0.140 | 0.32 | 0.15 | 0.67 | 0.003 | 0.11 | 0.03 | 0.37 | <0.001 |
| On most days but not every day | 0.25 | 0.15 | 0.41 | 0.000 | 0.38 | 0.20 | 0.74 | 0.004 | 0.13 | 0.06 | 0.29 | <0.001 | 0.14 | 0.04 | 0.47 | 0.001 |
| Everyday | 0.21 | 0.13 | 0.35 | 0.000 | 0.31 | 0.16 | 0.61 | 0.001 | 0.14 | 0.06 | 0.30 | <0.001 | 0.09 | 0.03 | 0.34 | 0.000 |
| **Broccoli, cabbage, collard greens** |  |  |  |  |  |  |  |  |  |  |  |  |  |  |  |  |
| Never or <1 time/month | 1 |  |  |  | 1 |  |  |  | 1 |  |  |  | 1 |  |  |  |
| 1 to 3 times/month | 0.63 | 0.39 | 1.03 | 0.066 | 0.91 | 0.49 | 1.66 | 0.751 | 0.47 | 0.22 | 1.00 | 0.049 | 0.19 | 0.05 | 0.68 | 0.010 |
| 1 to 2 times/week | 0.48 | 0.32 | 0.71 | <0.001 | 0.57 | 0.34 | 0.95 | 0.031 | 0.27 | 0.14 | 0.51 | <0.001 | 0.08 | 0.03 | 0.25 | <0.001 |
| On most days but not every day | 0.38 | 0.24 | 0.59 | <0.001 | 0.43 | 0.25 | 0.77 | 0.004 | 0.11 | 0.05 | 0.23 | <0.001 | 0.12 | 0.04 | 0.41 | 0.001 |
| Everyday | 0.24 | 0.14 | 0.41 | <0.001 | 0.34 | 0.18 | 0.66 | 0.001 | 0.08 | 0.03 | 0.19 | <0.001 | 0.08 | 0.02 | 0.35 | 0.001 |
| **Carrot** |  |  |  |  |  |  |  |  |  |  |  |  |  |  |  |  |
| Never or <1 time/month | 1 |  |  |  | 1 |  |  |  | 1 |  |  |  | 1 |  |  |  |
| 1 to 3 times/month | 0.58 | 0.38 | 0.88 | 0.011 | 0.91 | 0.54 | 1.54 | 0.736 | 0.43 | 0.22 | 0.84 | 0.013 | 4.31 | 1.25 | 14.84 | 0.021 |
| 1 to 2 times/week | 0.33 | 0.23 | 0.47 | <0.001 | 0.46 | 0.29 | 0.72 | 0.001 | 0.27 | 0.16 | 0.46 | <0.001 | 1.12 | 0.30 | 4.15 | 0.865 |
| On most days but not every day | 0.43 | 0.28 | 0.64 | <0.001 | 0.55 | 0.32 | 0.94 | 0.029 | 0.25 | 0.13 | 0.49 | <0.001 | 0.23 | 0.06 | 0.92 | 0.037 |
| Everyday | 0.37 | 0.22 | 0.62 | <0.001 | 1.04 | 0.55 | 1.97 | 0.906 | 0.22 | 0.09 | 0.52 | 0.001 | 0.64 | 0.16 | 2.60 | 0.530 |
| **Fresh fruit** |  |  |  |  |  |  |  |  |  |  |  |  |  |  |  |  |
| Never or <1 time/month | 1 |  |  |  | 1 |  |  |  | 1 |  |  |  | 1 |  |  |  |
| 1 to 3 times/month | 0.69 | 0.39 | 1.24 | 0.218 | 0.29 | 0.14 | 0.60 | 0.001 | 0.36 | 0.14 | 0.93 | 0.035 | 0.32 | 0.08 | 1.33 | 0.117 |
| 1 to 2 times/week | 0.31 | 0.18 | 0.53 | <0.001 | 0.23 | 0.12 | 0.42 | <0.001 | 0.18 | 0.08 | 0.41 | <0.001 | 0.17 | 0.05 | 0.63 | 0.008 |
| On most days but not every day | 0.21 | 0.12 | 0.36 | <0.001 | 0.23 | 0.12 | 0.44 | <0.001 | 0.15 | 0.07 | 0.34 | <0.001 | 0.08 | 0.02 | 0.34 | 0.001 |
| Everyday | 0.16 | 0.09 | 0.27 | <0.001 | 0.15 | 0.08 | 0.28 | <0.001 | 0.07 | 0.03 | 0.16 | <0.001 | 0.10 | 0.03 | 0.36 | <0.001 |
| **Fresh fruit juices** |  |  |  |  |  |  |  |  |  |  |  |  |  |  |  |  |
| Never or <1 time/month | 1 |  |  |  | 1 |  |  |  | 1 |  |  |  | 1 |  |  |  |
| 1 to 3 times/month | 0.80 | 0.54 | 1.19 | 0.272 | 0.45 | 0.27 | 0.76 | 0.003 | 0.44 | 0.25 | 0.80 | 0.006 | 0.84 | 0.29 | 2.40 | 0.744 |
| 1 to 2 times/week | 0.65 | 0.46 | 0.90 | 0.010 | 0.49 | 0.33 | 0.75 | 0.001 | 0.30 | 0.18 | 0.51 | 0.000 | 0.59 | 0.21 | 1.65 | 0.315 |
| On most days but not every day | 0.89 | 0.61 | 1.30 | 0.552 | 0.89 | 0.55 | 1.44 | 0.634 | 0.33 | 0.17 | 0.62 | 0.001 | 0.76 | 0.23 | 2.54 | 0.652 |
| Everyday | 0.57 | 0.37 | 0.88 | 0.011 | 0.60 | 0.36 | 0.99 | 0.044 | 0.27 | 0.14 | 0.52 | <0.001 | 1.67 | 0.60 | 4.62 | 0.326 |
| **Apples or pears** |  |  |  |  |  |  |  |  |  |  |  |  |  |  |  |  |
| Never or <1 time/month | 1 |  |  |  | 1 |  |  |  | 1 |  |  |  | 1 |  |  |  |
| 1 to 3 times/month | 0.35 | 0.24 | 0.51 | <0.001 | 0.34 | 0.21 | 0.54 | <0.001 | 0.22 | 0.12 | 0.41 | <0.001 | 0.25 | 0.09 | 0.66 | 0.005 |
| 1 to 2 times/week | 0.27 | 0.19 | 0.38 | <0.001 | 0.29 | 0.19 | 0.44 | <0.001 | 0.15 | 0.09 | 0.27 | <0.001 | 0.17 | 0.06 | 0.48 | 0.001 |
| On most days but not every day | 0.20 | 0.13 | 0.31 | <0.001 | 0.23 | 0.13 | 0.39 | <0.001 | 0.09 | 0.04 | 0.20 | <0.001 | 0.24 | 0.08 | 0.75 | 0.014 |
| Everyday | 0.15 | 0.09 | 0.26 | <0.001 | 0.15 | 0.07 | 0.32 | <0.001 | 0.09 | 0.04 | 0.20 | <0.001 | 0.30 | 0.08 | 1.07 | 0.063 |
| **Citrus fruit (oranges, lemons, tangerines)** |  |  |  |  |  |  |  |  |  |  |  |  |  |  |  |  |
| Never or <1 time/month | 1 |  |  |  | 1 |  |  |  | 1 |  |  |  | 1 |  |  |  |
| 1 to 3 times/month | 0.53 | 0.33 | 0.84 | 0.007 | 0.38 | 0.21 | 0.70 | 0.002 | 0.49 | 0.23 | 1.04 | 0.064 | 0.87 | 0.26 | 2.97 | 0.828 |
| 1 to 2 times/week | 0.30 | 0.20 | 0.46 | <0.001 | 0.38 | 0.23 | 0.65 | <0.001 | 0.46 | 0.23 | 0.90 | 0.024 | 0.39 | 0.12 | 1.31 | 0.128 |
| On most days but not every day | 0.19 | 0.12 | 0.30 | <0.001 | 0.25 | 0.14 | 0.44 | <0.001 | 0.21 | 0.10 | 0.45 | <0.001 | 0.42 | 0.12 | 1.47 | 0.177 |
| Everyday | 0.16 | 0.10 | 0.25 | <0.001 | 0.25 | 0.14 | 0.44 | <0.001 | 0.19 | 0.09 | 0.38 | <0.001 | 0.28 | 0.08 | 0.95 | 0.041 |
| **Fresh tomatoes** |  |  |  |  |  |  |  |  |  |  |  |  |  |  |  |  |
| Never or <1 time/month | 1 |  |  |  | 1 |  |  |  | 1 |  |  |  | 1 |  |  |  |
| 1 to 3 times/month | 0.45 | 0.25 | 0.81 | 0.008 | 0.55 | 0.24 | 1.23 | 0.143 | 0.38 | 0.15 | 0.97 | 0.042 | 0.00 | 0.00 |  | 0.999 |
| 1 to 2 times/week | 0.29 | 0.18 | 0.46 | <0.001 | 0.50 | 0.26 | 0.97 | 0.041 | 0.40 | 0.19 | 0.82 | 0.012 | 0.28 | 0.09 | 0.88 | 0.029 |
| On most days but not every day | 0.22 | 0.14 | 0.35 | <0.001 | 0.35 | 0.18 | 0.68 | 0.002 | 0.25 | 0.12 | 0.51 | <0.001 | 0.26 | 0.08 | 0.78 | 0.017 |
| Everyday | 0.15 | 0.09 | 0.25 | <0.001 | 0.41 | 0.21 | 0.80 | 0.009 | 0.21 | 0.10 | 0.42 | <0.001 | 0.17 | 0.06 | 0.52 | 0.002 |
| **Bananas** |  |  |  |  |  |  |  |  |  |  |  |  |  |  |  |  |
| Never or <1 time/month | 1 |  |  |  | 1 |  |  |  | 1 |  |  |  | 1 |  |  |  |
| 1 to 3 times/month | 0.83 | 0.45 | 1.52 | 0.539 | 0.33 | 0.15 | 0.74 | 0.007 | 0.68 | 0.26 | 1.81 | 0.441 | 0.22 | 0.04 | 1.37 | 0.105 |
| 1 to 2 times/week | 0.39 | 0.23 | 0.65 | <0.001 | 0.31 | 0.17 | 0.58 | <0.001 | 0.69 | 0.31 | 1.57 | 0.379 | 0.35 | 0.08 | 1.58 | 0.174 |
| On most days but not every day | 0.32 | 0.19 | 0.53 | <0.001 | 0.22 | 0.12 | 0.42 | <0.001 | 0.29 | 0.13 | 0.67 | 0.003 | 0.20 | 0.05 | 0.85 | 0.030 |
| Everyday | 0.16 | 0.10 | 0.27 | <0.001 | 0.18 | 0.10 | 0.34 | <0.001 | 0.20 | 0.09 | 0.44 | <0.001 | 0.15 | 0.04 | 0.60 | 0.007 |
| **Rice and beans** |  |  |  |  |  |  |  |  |  |  |  |  |  |  |  |  |
| Do not consume rice and beans or consume little | 1 |  |  |  | 1 |  |  |  | 1 |  |  |  | 1 |  |  |  |
| Consume rice and consume little beans | 0.45 | 0.21 | 0.94 | 0.033 | 0.46 | 0.16 | 1.31 | 0.147 | 1.27 | 0.38 | 4.22 | 0.697 | 0.57 | 0.03 | 11.85 | 0.718 |
| Consume little rice and consume more beans | 0.17 | 0.02 | 1.45 | 0.106 | 0.53 | 0.09 | 3.01 | 0.471 | 0.30 | 0.03 | 3.07 | 0.310 | 0.00 | 0.00 |  | 0.999 |
| Consume rice and beans almost daily | 0.68 | 0.41 | 1.14 | 0.147 | 0.64 | 0.34 | 1.18 | 0.153 | 0.72 | 0.29 | 1.81 | 0.490 | 1.43 | 0.15 | 13.13 | 0.754 |

OR, odds ratio; CI, confidence interval; BMI, body mass index.
